# Use of HuH6 and other human-derived hepatoma lines for the detection of genotoxins: a new hope for laboratory animals?

**DOI:** 10.1007/s00204-017-2109-4

**Published:** 2017-12-07

**Authors:** Monika Waldherr, Miroslav Mišík, Franziska Ferk, Jana Tomc, Bojana Žegura, Metka Filipič, Wolfgang Mikulits, Sören Mai, Oskar Haas, Wolfgang W. Huber, Elisabeth Haslinger, Siegfried Knasmüller

**Affiliations:** 1 Department of Internal Medicine I, Institute of Cancer Research, Medical University Vienna, Borschkegasse 8a, 1090 Vienna, Austria; 2 Department for Genetic Toxicology and Cancer Biology, National Institute of Biology, Večna pot 111, Ljubljana, Slovenia; 3 Jozef Stefan International Postgraduate School, Jamova cesta 39, 1000 Ljubljana, Slovenia; 4 Labdia Labordiagnostik GmbH, Zimmermannplatz 8, 1090 Vienna, Austria

**Keywords:** Hepatic cell lines, p53, Comet assay, Genotoxicity

## Abstract

Cell lines which are currently used in genotoxicity tests lack enzymes which activate/detoxify mutagens. Therefore, rodent-derived liver preparations are used which reflect their metabolism in humans only partly; as a consequence misleading results are often obtained. Previous findings suggest that certain liver cell lines express phase I/II enzymes and detect promutagens without activation; however, their use is hampered by different shortcomings. The aim of this study was the identification of a suitable cell line. The sensitivity of twelve hepatic cell lines was investigated in single cell gel electrophoresis assays. Furthermore, characteristics of these lines were studied which are relevant for their use in genotoxicity assays (mitotic activity, p53 status, chromosome number, and stability). Three lines (HuH6, HCC1.2, and HepG2) detected representatives of five classes of promutagens, namely, IQ and PhIP (HAAs), B(a)P (PAH), NDMA (nitrosamine), and AFB_1_ (aflatoxin), and were sensitive towards reactive oxygen species (ROS). In contrast, the commercially available line HepaRG, postulated to be a surrogate for hepatocytes and an ideal tool for mutagenicity tests, did not detect IQ and was relatively insensitive towards ROS. All other lines failed to detect two or more compounds. HCC1.2 cells have a high and unstable chromosome number and mutated p53, these features distract from its use in routine screening. HepG2 was frequently employed in earlier studies, but pronounced inter-laboratory variations were observed. HuH6 was never used in genotoxicity experiments and is highly promising, it has a stable karyotype and we demonstrated that the results of genotoxicity experiments are reproducible.

**Electronic supplementary material:**

The online version of this article (10.1007/s00204-017-2109-4) contains supplementary material, which is available to authorized users.

## Introduction

One of the fundamental problems of in vitro tests is the inadequate representation of drug metabolizing enzymes in cell lines which are currently used in routine screening of chemicals. Therefore, exogenous liver-derived enzyme homogenates are prepared from rodents which have been treated with enzyme inducers (Kirkland 1990). The homogenates contain phase I enzymes that convert chemicals to genotoxic metabolites and are added in experiments with bacteria and mammalian cells to mimic the biotransformation of chemicals in humans (Brandon et al. 2003). These experimental models do not reflect the situation in vivo, for example, the detoxification of electrophilic DNA reactive intermediates by phase II enzymes. As a consequence, often false results are obtained and animal experiments with rodents are performed which could be avoided with more reliable in vitro models (Kirkland et al. 2007). To develop more reliable in vitro tests, attempts were made to improve the sensitivity and specificity of existing tests (Fowler et al. 2012) and to establish new systems. The latter approaches include the development of 3D models, the immortalisation of primary human liver cells, the insertion of genes encoding for drug metabolizing enzymes in currently used cells, and attempts to find lines which have retained the activities of drug metabolizing enzymes (for reviews see Brandon et al. 2003; Donato et al. 2013; Zeilinger et al. 2016). Attempts to use primary hepatocytes and 3D cultures were only partly successful as they are costly and time consuming while lines expressing individual drug metabolizing enzymes are useful for mechanistic studies but not for routine testing.

In the early 1990s, Darroudi and Natarajan (1991) showed that the human-derived hepatoma line HepG2 detects representatives of many groups of genotoxic procarcinogens without addition of exogenous rodent-derived enzyme homogenate. Their findings were confirmed in subsequent experiments and it was shown that this line is able to identify DNA reactive carcinogens which give false negative results in conventional in vitro tests. Furthermore, it was found that these cells can discriminate between structurally related mutagens and non-mutagens (for review see Knasmuller et al. 1998). The cells possess a variety of drug metabolizing phase I and II enzymes in inducible form, which play a role in the activation and detoxification of chemical mutagens. Therefore, HepG2 cells were also considered as a suitable tool for the detection of synergistic and antagonistic effects in complex mixtures (Mersch-Sundermann et al. 2004). However, comparison of results obtained with HepG2 cells in different labs showed that the sensitivity of this line varies strongly (Knasmuller et al. 2004). One of the reasons for the poor reproducibility of experiments with these cells may be strong variations of the transcription of drug metabolizing enzymes (Wilkening et al. 2003). In the following years, a few other human hepatoma cell lines were identified which express xenobiotic drug metabolizing enzymes and can be used to detect promutagens. These lines include Hep3B (Majer et al. 2004), HCC1.2 (Winter et al. 2008) and HepaRG (Le Hegarat et al. 2014). Recently, it was postulated that the latter line is more suitable for mutagenicity and biotransformation studies than HepG2 cells (Le Hegarat et al. 2014); however, the majority of experimental data comes from one laboratory and further confirmation of this assumption is required.

The use of the different human-derived liver cells in mutagenicity tests was not based on targeted comparative screening of currently available cell lines and we hypothesized that other lines which are currently available may be equally or even more suitable for the detection of mutagens. Therefore, we performed a comprehensive study in which we compared the sensitivity of a panel of cell lines towards representatives of different classes of genotoxic carcinogens which require enzymatic activation in genotoxicity experiments. In addition to HepG2, Hep3B, HCC1.2 and HepaRG, eight lines were included which have never been used in genotoxicty assays before. The test compounds which were used are listed in Table 1; all of them are of human relevance and require metabolic activation by different phase I and phase II enzymes. Hydrogen peroxide (H_2_O_2_) was included in all experimental series as a positive control, this compound is directly active and causes damage of the DNA via formation of reactive oxygen species (ROS).

**Table 1:** Occurrence, mutagenicity, carcinogenic properties and metabolism of test compounds

Chemical (abbreviation), occurrence	Chemical group	Mutagenic and carcinogenic properties	Metabolism	References
Aflatoxin B1 (AFB_1_), produced by A. flavus and A. parasiticus; in peanuts, maize and cottonseeds	Mycotoxins	Causes formation of epoxide induce risk for HCC in humans and animals, clear evidence for carcinogenicity IARC: Group 1	Activation by CYP1A2, 2B6, 3A4, 3A5, 3A7; inhibition of DNA-adduct formation by GSTs	(IARC 1993)
Benzo(a)pyrene (B(a)P), tobacco smoke, ambient air, grilled/broiled and smoke-cured meats, vegetables grown in contaminated soils	Polycyclic aromatic hydrocarbons (PAH)	Causes guanine adducts and tumors in many animal species, local and systemic carcinogenic effects IARC: Group 1	Epoxide formation via CYP1A1, 1A2 and 1B1, conjugation of metabolites with GSTs, UGTs and SULTs	(IARC 2010)
2-Amino-3-methyl-3H-imidazo[4,5-f]quinolone (IQ), grilled/boiled meat and fish	Heterocyclic aromatic amines (HAA)	Causes guanine adducts, clear evidence for carcinogenicity in experiments with rodents IARC: Group 2A	Activation to DNA reactive species by CYP1A2 and NAT detoxification via GSTs and SULTs	(IARC 1993)
2-Amino-1-methyl-6-phenylimidazo[4,5-b]pyridine (PhIP), cooked beef, pork, chicken and fish products	HAA	Causes guanine adducts that induces tumors at multiple sites in animal models IARC: Group 2B	Activation through CYP1A2 and further metabolism by SULTs, detoxification via UGTs	(IARC 1993)
N-Nitroso-dimethylamine (NDMA), tobacco smoke, meat and fish products, endogenous formation (stomach)	Nitrosamines	Causes DNA methylation and induction of cancer in liver, lungs and kidneys of mice and of HCC in rats IARC: Group 2A	Activation by CYP2E1, unstable metabolite dissociates to DNA reactive compound	(IARC 1978)
Hydrogen peroxide (H_2_O_2_)	Peroxides	Causes oxidation, directly acting mutagen IARC: Group 3	No activation required; decomposition leads to formation OH^**·**^ and O_2_^**·**^ radicals	(Menghini 1988)

*HCC*, human hepatoma cell carcinoma; *GST*, Glutathione S-transferases; *NAT*, *N*-acetyltransferase; *SULTs*, sulfotransferase; *UGT* glucuronyltransferase

The DNA damaging properties of the different compounds were monitored in single cell gel electrophoresis (SCGE) assays which are based on the determination of DNA damage in an electric field and are increasingly used in genetic toxicology (Azqueta and Collins 2013; Collins 2015).

Additionally, we studied characteristics of the different cell lines which are relevant in regard to their potential use in genotoxicity tests, namely, (1) the morphology of the cells which provides information about their origin and similarity to primary liver cells, (2) their karyotype allowing to draw conclusions concerning their chromosomal stability, (3) measurements of the mitotic activities providing information about the duration of the repair phase which is needed for the design of tests which require cell division (e.g., gene mutation and micronucleus tests) and (4) the *p53* status of the cells as it was found that it plays an important role in regard to the reliability of results obtained with mammalian cell lines in mutagenicity experiments (Kirkland et al. 2007; Pfuhler et al. 2011).

## Materials and methods

### Chemicals

Citric acid (CAS-No. 77-92-9) and di-natriumhydrogenphosphate-dihydrate (CAS-No. 10028-24-7) for McIlvaine buffer were obtained from Merck (Darmstadt, Germany). Quinacrine dihydrochloride (CAS-No. 69-05-6) and Thymol (CAS-No. 89-83-8) for quinacrine staining solution, aflatoxin B_1_ (AFB_1_, CAS-No. 1162-65-8), benzo(a)pyrene (B(a)P, CAS-No. 50-32-8), hydrogen peroxide (H_2_O_2_, CAS-No. 7722-84-1) and *N*-nitrosodimethylamine (NDMA, CAS-No. 62-75-9) were purchased from Sigma-Aldrich (St Louis, Missouri, USA). 2-amino-3-methyl-3H-imidazo[4,5-f]quinolone (IQ, CAS-No. 76180-96-6) came from Toronto Research Chemicals (Toronto, Ontario, Canada) and 2-amino-1-methyl-6-phenylimidazo[4,5-b]pyridine (PhIP, CAS-No. 105650-23-5) from Santa Cruz Biotechnology, Inc. (Dallas, Texas, USA). Anti-GADPH antibody (ab9485), anti-mutant-p53 antibody (ab32049) and anti-p53 antibody (ab1101) were purchased from Abcam plc (Cambridge, UK). Anti-rabbit IgG HRP conjugate (W4018), anti-mouse IgG HRP (W4028) conjugate came from Promega Corporation (Madison, WI, USA).

Low melting point agarose (LMPA) and normal melting point agarose (NMPA) were acquired from Gibco (Paisley, UK). Inorganic salts, dimethyl sulfoxide (DMSO), propidium iodide, hydrogen peroxide, Triton X-100, Trizma base, fetal bovine serum (FBS), Dulbecco’s Phosphate Buffered Saline (DPBS), Dulbecco’s modified Eagle Medium (DMEM), Eagle’s Minimum Essential Medium (EMEM), Minimal essential Medium Eagle (MEME), Roswell Park Memorial Institute Medium (RPMI-1640) were purchased from Sigma-Aldrich (Steinheim, Germany). William’s E medium came from Thermo Fisher Scientific (Vienna, Austria). Trypsin–EDTA was ordered from Life Technologies (Karlsruhe, Germany).

### Cell lines

The origin of the cell lines is listed in Table 2 which provides also information on the cultivation conditions.

**Table 2:** Origin and cultivation of the cell lines

Cell line	Provider	Origin of line	Cultivation^a^	Reference
HCC 1.2	M. Eisenbauer (Institute of Cancer Research, MUW, Vienna, Austria)	Hepatocellular carcinoma of a 56-year-old male from the General Hospital of Vienna	RPMI-1640, 2.0 g/L NaHCO_3_	(Sagmeister et al. 2008)
Hep3B	ATCC (Manassas, VA, USA)	Hepatocellular carcinoma of a 8-year-old black male from the United States	EMEM, 2.2 g/L NaHCO_3_	(Aden et al. 1979)
HepaRG™	Thermo Fisher Scientific (Waltham, Massachusetts, USA)	Liver tumor of a female patient suffering from hepatitis C in France	William’s E medium	(Aninat et al. 2006)
HepG2	ATCC (Manassas, VA, USA)	Hepatocellular carcinoma of a 15-year-old Caucasian male from Argentina	MEME, 2.2 g/L NaHCO_3_, 1% NEAA, 1 mM CH_3_COCOONa	(Aden et al. 1979)
HuH6	Isabel Fabregat (IDIBELL, Barcelona, Spain)	Hepatoblastoma of a one-year-old Japanese boy	RPMI-1640, 2.0 g/L NaHCO_3_	(Doi 1976)
HuH7	Isabel Fabregat (IDIBELL, Barcelona, Spain)	Well-differentiated hepatocellular carcinoma of a 75-year-old Japanese male	DMEM, 3.7 g/L NaHCO_3_	(Clayton et al. 2005)
JHH6	Gabriele Grassi (Department of Life Sciences, University Hospital of Cattinara, Trieste, Italy)	Hepatocellular carcinoma of a 57-year-old Japanese female	William’s E medium, 1% glutamine	(Grassi et al. 2007)
PLC/PRF	ATCC (Manassas, VA, USA)	Primary liver carcinoma of a 24-year-old Shangaan male	MEME, 2.2 g/L NaHCO_3_	(Macnab et al. 1976)
SK-Hep1	ATCC (Manassas, VA, USA)	Adenocarcinoma of a 52-year-old Caucasian male	EMEM, 2.2 g/L NaHCO_3_	(Heffelfinger et al. 1992)
SNU-398	Isabel Fabregat (IDIBELL, Barcelona, Spain)	Hhepatocellular carcinoma of a Korean (42 years, male) patient	RPMI-1640, 2.0 g/L NaHCO_3_	(Park et al. 1995)
SNU-449	Isabel Fabregat (IDIBELL, Barcelona, Spain)	Hepatocellular carcinoma of a Korean (52 years, male) patient	RPMI-1640, 2.0 g/L NaHCO_3_	(Park et al. 1995)
WRL68	Isabel Fabregat (IDIBELL, Barcelona, Spain)	Spontaneous transformation from human embryonic liver tissue	DMEM, 3.7 g/L NaHCO_3_	(Gutierrezruiz et al. 1994)

*ATCC* American Type Culture Collection, *DMEM* Dulbecco’s Modified Eagle Medium, *EMEM* Eagle’s Minimum Essential Medium, *IDIBELL* Instituto de Investigación Biomédica de Bellvitge, *MEME* Minimal Essential Medium Eagle, *MUW* Medizinische Universität Wien, *NEAA* Non-Essential Amoni Acids, *RPMI* Roswell Park Memorial Institute Medium

^a^HuH6 cells were grown in 4.0% FBS, all other lines were grown in 10.0% FBS, and HCC 1.2 and JHH6 were cultivated in heat-inactivated FBS (10.0%). All lines were cultivated in 5.0% CO_2_, except HuH7 and WRL68 (both 8.0% CO_2_). HepaRG™ cells were cultivated as described by the provider (reconstitution with 1.0% glutamine, for thawing: 5 × HepaRG™ Thaw, Plate and General Purpose Medium Supplement, for passaging and toxicological experiments: 5 × HepaRG™ ToxMed Supplement)

All lines were cultivated at 37 °C, 96.0% humidity in 5.0 or 8.0% CO_2_. All lines except HepaRG™ were grown in TC-treated flasks and dishes (Sigma-Aldrich, St Louis, Missouri, USA) as described in previous publications (for details see Table 2) and were routinely checked for mycoplasma contaminations by PCR (Mycoplasma Plus PCR Primer Set, Cat. No. 302008, Agilent Technologies, Santa Clara, CA, USA). HepaRG™ cells were cultivated and used according to the instructions of the manufacturer (Thermo Fisher Scientific, Vienna, Austria).

Flasks and dishes for experiments with HepaRG™ were coated with Collagen R. Collagen R stock solution (Serva Electrophoresis GmbH, Heidelberg, Germany) was diluted to obtain a final concentration of 0.2 mg/ml collagen. Subsequently, the growth areas of the flasks or dishes were covered with 0.1 ml/cm^2^ of this solution for 30–40 min, subsequently collagen R solution was removed, then the flasks or dishes were air dried.

The identity of the cell lines (except of HepaRG™) was verified by short tandem repeat (STR) analyses (van Zijl et al. 2011).

From all lines, except HepaRG™, cryopreserved cultures were made; cells from confluent T75 flasks were harvested and resuspended after centrifugation (200 g, 5 min, 21 °C) in 5.0 ml medium. DMSO was added drop-wise (final concentration 5.0%), then the suspensions were distributed equally to cryovials (Carl Roth, Karlsruhe, Germany), which were placed in a polystyrene box at − 80 °C for 24 h (to ensure slow freezing) and transferred to liquid nitrogen.

### Proliferation kinetics

The proliferation kinetics and size of the cells were monitored by use of a CASY^®^ Cell Counter and Analyzer System (TTC-2EA-1087, Schärfe System GmbH, Reutlingen, Germany) which was used as described by the manufacturer (Schärfe-System GmbH, Reutlingen, Germany). Cell lines were grown in Petri dishes (Ø 6 cm, Sigma-Aldrich, St Lois, Missouri, USA) in 5.0 ml medium; ca. 5 × 10^5^ cells were seeded in each dish at the start of the experiments. After 24 h intervals, the cells were detached with trypsin–EDTA (Szabo-Scandic, Vienna, Austria) and 50 µl of these suspensions were transferred to CASY-cups (OLS OMNI Life Science GmbH & Co. KG, Bremen, Germany). For each experimental point, three plates were evaluated. On the basis of the results, means and standard deviations (SDs) were calculated. Nonlinear fits with exponential growth equations (least square fits) were calculated to determine the doubling times (http://www.graphpad.com/guides/prism/5/user-guide/prism5help.html?reg_exponential_growth.htm).

### Karyotyping

Cell lines were cultured and prepared according to standard cytogenetic techniques; at least 20 metaphases per cell line were karyotyped. Slides, which had been stained with quinacrine solution (Sigma-Aldrich, St Louis, Missouri, USA), were incubated in McIlvaine solution (Merck, Darmstadt, Germany), covered with a cover slip and analyzed using Applied Spectral Imaging Case Data Manager (Version 5.5.2.2, Applied Spectral Imaging, Carlsbad, CA, USA).

### Determination of the *p53* status

The expression of the *p53* gene in the different cell lines was analyzed using real-time quantitative PCR (RT-qPCR) according to the description of Straser et al. (2013). The protein was quantified by Western blotting as described by Pezdirc et al. (2013). Furthermore, the induction of p53 protein and of mRNA were investigated in all experiments after incubation of the cell lines with 30 µM B(a)P.

### SCGE assays

The SCGE experiments were conducted as described in an international guideline (Tice et al. 2000). Reagents for the assays (lysis solution and alkaline electrophoresis buffer) and agarose-coated slides were prepared according to Collins and Dusinska (2009).

Briefly, cells were sub-cultured in 1.0 ml medium in 24–well tissue-culture plates (Sarstedt Inc., Newton, NC, USA). After 24 h, the media were changed and the cells exposed to different concentrations of the test compounds or to the solvent controls (PBS or DMSO). H_2_O_2_ and NDMA were dissolved in PBS (pH 7.0), all other compounds in DMSO. The final DMSO concentrations in the media did not exceed 1.0%. The exposure time in experiments with AFB_1_, B(a)P and NDMA was 24 h and in assays with PhIP and IQ 48 h. To terminate the treatment, the cells were washed twice with PBS (1.0 ml), trypsinized (100 µl T/E per well, 4 min) and then resuspended in 400 µl culture medium containing FBS. Subsequently, the suspensions were transferred to Eppendorf tubes and centrifuged (200*g*, 5 min). Cell pellets were resuspended in cold PBS. H_2_O_2_ treatment was conducted with cells embedded on the slides by exposure to different concentrations (10–50 µM) of cold H_2_O_2_ solution in PBS for 5 min, followed by washing with cold PBS (5 min).

In all experiments, acute toxic effects were determined with a CASY^®^ Cell Counter and Analyzer System (Schärfe-System GmbH, Reutlingen, Germany) (Lindl et al. 2005). SCGE assays were only performed when the viability of the cultures was ≥ 70% (Koppen et al. 2017). Per experimental point, two cultures were set up in parallel and from each one slide with two separate gels (30,000 cells/gel) was prepared. After unwinding (40 min, pH > 13) and electrophoresis (30 min, 0.75 V/cm, ~ 300 mA, 4 °C, pH > 13), 50 cells were evaluated per gel (200 cells per experimental point). As an endpoint, we determined the tail intensities (“% DNA in tail”) with a computer-aided system Comet Assay IV (Perceptive Instruments, Bury St Edmunds, UK), which is at present the most widely used parameter (Collins et al. 2008; Moller 2006). For each gel, the median of the tail intensity was calculated.

### Statistical analyses

The results of the genotoxicity screening experiments were analyzed using GraphPad Prism 5 to perform ordinary one-way ANOVA followed by Dunnett’s multiple comparisons. The results of the RT-qPCR analyses were evaluated with the ΔΔCt method; GAPDH was used as a reference gene. *P* values ≤ 0.05 were considered as statistically significant.

## Results

### General characteristics of the liver cell lines

Figure S1 shows morphology of the different cell types, their morphological characteristics are described in Table 3, which contains also information concerning their size, which was in a relatively narrow range (i.e., between 16 and 22 µm). In agreement with literature data (see Table 2), we found that most lines have an epithelial morphology, however, some cell types (SNU-398, SNU-449, SK-Hep1 and WRL68) had mesenchymal features and most of them (except SK-Hep1) had a polygonal form.

**Table 3:** Characterization of the morphology, growth kinetics, karyotype and *p53*-status of the different cell lines

Cell line	Morphology	Doubling time (h)	Chromosome number^a^	P53 protein expression (Western Blot)^b^		P53 gene expression (RT-qPCR)^c^		Size (µm)
				Background	Induced^d^	Background	Induced^e^	
HCC 1.2	Epithelial	46–53	111–127	**+**	**+**	1	1.50 ± 0.33	Ø 22
Hep3B	Epithelial liver parenchymal	41–53	62–67	−	−	0	0	Ø 19
HepaRG™	Epithelial granular hepatocyte-like	48–69	46–47	**+**	**+**	1	0.87 ± 0.14	Ø 20
HepG2	Epithelial resemble liver parenchym	41–57	49-53	**+**	**+++**	1	1.48 ± 0.18	Ø 18
HuH6	Epithelial desmosomes and glycogen granules in the cytoplasm	45–50	82–86	**+**	**++**	1	1.26 ± 0.01	Ø 23
HuH7	Epithelial, grow in multilayered islands, often piled up, peripheral cells surrounding the island appeared to be flattened	23–27	65–111	**+**	**+**	1	1.13 ± 0.01	Ø 22
JHH6	Epithelial undifferentiated morphology	33–47	53–70	**+**	**+**	1	1.00 ± 0.03	Ø 19
PLC/PRF	Epithelial polygonal in shape with well-defined borders, many binucleated cells	36–43	39–58	**+**	**+**	1	0.89 ± 0.05	Ø 20
SK-Hep1	Mesenchymal, HCC-like cell shape	35–54	59–61	**+**	**+**	1	1.04 ± 0.12	Ø 19
SNU-398	Mesenchymal round-spindle, multinuclear, trabecular arrangements, anaplastic small cells	30–33	59–64	**+**	**+**	1	1.03 ± 0.15	Ø 16
SNU-449	Mesenchymal polygonal, single or double nuclear cells, compact growth pattern, trabecular	28–33	52–55	**+**	**+**	1	0.95 ± 0.03	Ø 21
WRL68	Mesenchymal polygonal to spindle shape, some cells rounded, morphology similar to human hepatocytes	25–30	61–254	**+**	**+**	1	0.96 ± 0.03	Ø 20

^a^Analysis of 50 quinacrine-stained metaphase spreads

^b^In HCC 1.2 and SNU-398 mutated p53 was detected with an anti-mutant-p53 antibody

^c^TaqMan^®^ Gene Expression Assay, TP53 Hs00153349_m1, AB (Coverage: 7 transcripts for p53), for Hep3B, HepaRG™, JHH6 and SNU-449 TaqMan^®^ Gene Expression Assay, TP53 Hs01034249_m1, AB (Coverage: 15 transcripts for p53) was used

^d^Induction of p53-protein expression was monitored after treatment with 30 µM B(a)P for 24 h. No expression is indicated with −, background expression with +, induced expression with ++ and highly induced expression with +++

^e^Statistically significant induction of gene expression after treatment with 30 µM B(a)P for 24 h is indicated with asterisks

The features of HuH6 cells differ from those of the other cell types, i.e., they are relatively undifferentiated and we found in agreement with previous studies that they contain numerous glycogen granules, which are only rarely seen in other liver-derived cells (Figure S1E) (Doi 1976).

The proliferation kinetics of the different cells are shown graphically in Figures S2a and b. On the basis of the experimental data, the doubling times were calculated (Table 3). The “fastest” lines were HuH7 and WRL68 (23–27 h and 25–30 h, respectively), relatively slow mitotic activities were found with HepaRG and HuH6 cells (48–69 h and 45–50 h).

The results of the karyotyping experiments are summarized in column four of Table 3. It can be seen that the chromosome numbers and also the range varied substantially. High numbers (i.e. more than 100 chromosomes per cell) were detected in HCC1.2, HuH7 and WRL68. The numbers in HepaRG and HepG2 are similar to those found in primary human hepatocytes. Figure S3 shows a representative karyogram of HuH6 cells. The typical number of chromosomes in this cell line was between 82 and 86. In some lines, the number of chromosomes was in a narrow range (SK-Hep1, Hep3B, HepaRG, and HuH6) while a broader range was found in other cell types (e.g., in WRL68).

Western Blot and RT-qPCR analyses (Table 3; Figure S4) revealed that Hep3B cells lack p53 expression at the transcriptional and at the protein level while HCC1.2 and SNU-398 possess mutated p53 protein. The protein was induced in HepG2 and HuH6 after B(a)P treatment for 24 h.

### Sensitivity of the different hepatic cell lines towards model mutagens

The results which were obtained in representative SCGE experiments with the different liver lines are summarized in Table 4. It can be seen that the responses varied strongly. Five lines (PLC/PRF, SK-Hep1, SNU-398, SNU-449, and WRL68) were not responsive to the different model compounds. In HuH7, induction of DNA damage was found with high doses of B(a)P and NDMA but not with the other genotoxins. Hep3B cells were in general insensitive but a moderate effect was obtained with B(a)P. HepaRG cells detected four out of five genotoxins (all compounds except IQ). In HCC1.2, HepG2, and HuH6 cells, all compounds induced positive results. It is also notable that the latter line and HCC1.2 cells were the most sensitive ones, i.e., positive results were observed with most mutagens at relatively low concentrations.

**Table 4:** Induction of DNA migration in the human derived liver cell lines by different genotoxins

Cell line	AFB_1_		B(a)P		IQ		NDMA		PhIP	
	Conc. (µM)	% DNA in tail	Conc. (µM)	% DNA in tail	Conc. (µM)	% DNA in tail	Conc. (mM)	% DNA in tail	Conc. (µM)	% DNA in tail
HCC 1.2	0.0 1.0 5.0 20.0	1.38 ± 0.48 3.74 ± 2.69 4.02 ± 1.22 8.75 ± 1.48*	0.0 2.0 10.0 50.0	0.68 ± 0.50 2.28 ± 1.77 3.07 ± 0.97 5.69 ± 2.10*	0.0 25.0 50.0 100.0	2.93 ± 1.40 1.39 ± 0.94 1.86 ± 0.44 7.29 ± 1.87*	0.0 50.0 100.0 200.0	1.77 ± 1.64 16.3 ± 4.52* 17.1 ± 3.64* 8.87 ± 0.30*	0.0 50.0 100.0 300.0	6.61 ± 2.14 17.5 ± 4.84* 15.1 ± 2.32* 14.8 ± 2.63*
Hep3B	0.0 1.0 5.0 20.0	0.93 ± 0.86 1.35 ± 0.81 4.03 ± 1.93* 4.31 ± 0.92*	0.0 10.0 30.0 90.0	0.79 ± 0.66 3.97 ± 1.51* 2.47 ± 1.77 2.80 ± 1.30	0.0 50.0 100.0 250.0	0.92 ± 0.58 0.88 ± 0.71 1.75 ± 1.45 2.09 ± 1.38	0.0 50.0 100.0 200.0	0.57 ± 0.21 0.62 ± 0.45 0.92 ± 0.45 0.61 ± 0.47	0.0 25.0 50.0 100.0	1.56 ± 0.18 1.18 ± 0.39 1.43 ± 0.24 0.82 ± 0.53
HepaRG	0.0 5.0 10.0 20.0	4.16 ± 1.40 11.6 ± 1.50* 9.67 ± 2.17* 7.5 ± 1.76*	0.0 2.0 10.0 50.0	1.43 ± 1.54 0.79 ± 0.36 5.72 ± 0.96* 6.71 ± 2.24*	0.0 25.0 50.0 100.0	2.34 ± 2.92 3.34 ± 3.31 2.39 ± 4.10 2.38 ± 1.97	0.0 50.0 100.0 200.0	0.96 ± 0.59 1.47 ± 0.10 1.41 ± 1.04 5.09 ± 0.52*	0.0 25.0 50.0 100.0	0.53 ± 0.66 2.56 ± 1.95 4.82 ± 3.57* 2.86 ± 1.73
HepG2	0.0 1.0 5.0 20.0	1.02 ± 0.28 0.85 ± 0.41 2.96 ± 0.66* 5.17 ± 1.57*	0.0 10.0 30.0 90.0	0.83 ± 0.62 14.3 ± 3.03* 15.4 ± 2.53* 13.5 ± 2.79*	0.0 50.0 100.0 250.0	2.16 ± 0.54 1.32 ± 0.91 5.38 ± 2.02* 5.39 ± 1.94*	0.0 50.0 100.0 200.0	0.35 ± 0.21 0.83 ± 0.26 1.12 ± 0.72 1.55 ± 0.49*	0.0 25.0 50.0 100.0	2.90 ± 1.03 1.33 ± 0.83 6.92 ± 2.00* 6.78 ± 1.37*
HuH6	0.0 10.0 20.0 30.0	1.50 ± 1.09 3.35 ± 2.75 7.20 ± 3.1* 11.9 ± 4.30*	0.0 10.0 30.0 90.0	0.91 ± 0.49 3.54 ± 0.88* 7.44 ± 1.58* 5.16 ± 1.91*	0.0 25.0 50.0 100.0	4.01 ± 1.68 5.49 ± 2.24 5.91 ± 3.05 10.82 ± 5.71*	0.0 50.0 100.0 200.0	1.73 ± 2.62 2.91 ± 1.45 6.86 ± 1.74* 13.1 ± 4.41*	0.0 50.0 100.0 300.0	1.81 ± 1.15 11.51 ± 3.64* 8.67 ± 2.67* 24.7 ± 2.15*
HuH7	0.0 1.0 2.5 5.0	6.29 ± 1.57 2.76 ± 0.52 3.12 ± 0.70 5.81 ± 4.64	0.0 10.0 30.0 90.0	1.08 ± 0.47 2.00 ± 1.14 4.79 ± 1.25* 5.36 ± 1.03*	0.0 50.0 100.0 250.0	1.29 ± 1.30 2.16 ± 0.91 1.11 ± 0.60 1.88 ± 1.68	0.0 50.0 100.0 200.0	0.61 ± 0.35 0.69 ± 0.48 0.77 ± 0.60 3.01 ± 2.11*	0.0 50.0 100.0 300.0	5.12 ± 1.05 5.35 ± 2.84 9.21 ± 2.26 3.55 ± 2.08
JHH6	0.0 1.0 2.5 5.0	1.67 ± 0.99 1.7 ± 0.75 n.d. 4.52 ± 1.43*	0.0 10.0 50.0 90.0	1.67 ± 0.99 2.33 ± 1.18 3.78 ± 3.42 3.37 ± 1.96	0.0 25.0 50.0 100.0	0.59 ± 0.21 0.76 ± 0.56 1.29 ± 1.55 0.93 ± 0.67	0.0 50.0 100.0 200.0	1.17 ± 1.11 1.33 ± 0.66 1.69 ± 0.81 1.16 ± 0.53	0.0 25.0 50.0 100.0	0.59 ± 0.21 0.25 ± 0.15 0.93 ± 1.09 1.44 ± 0.53
PLC/PRF	0.0 1.0 2.5 5.0	1.84 ± 1.44 4.57 ± 1.83 4.77 ± 1.75 6.68 ± 2.94*	0.0 10.0 30.0 90.0	1.09 ± 0.81 1.39 ± 0.55 1.31 ± 1.72 1.54 ± 0.87	0.0 50.0 100.0 250.0	2.47 ± 2.08 5.17 ± 4.50 2.84 ± 2.65 4.61 ± 1.54	0.0 50.0 100.0 200.0	1.15 ± 1.25 1.04 ± 0.33 1.65 ± 1.55 1.09 ± 0.33	0.0 50.0 100.0 300.0	3.82 ± 1.22 2.51 ± 0.56 4.00 ± 3.72 2.43 ± 2.03
SK-Hep1	0.0 1.0 5.0 20.0	0.60 ± 0.41 1.30 ± 0.37 1.13 ± 0.72 1.24 ± 0.88	0.0 10.0 30.0 90.0	0.46 ± 0.29 0.87 ± 0.86 2.20 ± 2.36 1.17 ± 0.80	0.0 50.0 100.0 250.0	0.59 ± 0.24 0.62 ± 0.47 1.43 ± 0.95 0.68 ± 0.70	0.0 50.0 100.0 200.0	0.46 ± 0.16 0.70 ± 0.42 0.59 ± 0.24 0.37 ± 0.34	0.0 50.0 100.0 300.0	2.84 ± 2.06 5.18 ± 1.19 5.83 ± 1.06 5.77 ± 4.30
SNU-398	0.0 1.0 5.0 10.0	2.50 ± 1.08 2.53 ± 1.68 3.06 ± 0.28 2.83 ± 0.61	0.0 10.0 30.0 90.0	0.64 ± 0.27 0.73 ± 0.45 0.56 ± 0.45 2.28 ± 2.73	0.0 50.0 100.0 250.0	1.02 ± 0.88 1.42 ± 0.72 1.20 ± 0.81 1.63 ± 1.25	0.0 50.0 100.0 200.0	0.40 ± 0.14 0.65 ± 0.50 0.83 ± 0.53 0.28 ± 0.11	0.0 50.0 100.0 300.0	5.60 ± 3.26 2.92 ± 1.18 7.04 ± 5.97 10.3 ± 1.06
SNU-449	0.0 1.0 2.5 5.0	2.87 ± 2.05 2.04 ± 2.18 1.14 ± 1.95 3.37 ± 3.14	0.0 10.0 30.0 90.0	0.79 ± 0.41 1.26 ± 0.62 0.79 ± 0.21 0.74 ± 0.64	0.0 50.0 100.0 250.0	1.20 ± 1.18 3.67 ± 2.95 1.46 ± 1.21 0.18 ± 0.09	0.0 50.0 100.0 200.0	0.23 ± 0.16 0.52 ± 0.24 0.54 ± 0.11 0.15 ± 0.03	0.0 50.0 100.0 300.0	2.12 ± 0.59 2.02 ± 0.81 3.06 ± 1.08 1.17 ± 0.83
WRL68	0.0 1.0 2.5 5.0	3.14 ± 1.66 2.32 ± 0.89 1.98 ± 0.41 1.72 ± 0.78	0.0 10.0 30.0 90.0	2.66 ± 1.17 3.10 ± 3.43 4.30 ± 0.34 6.10 ± 3.26	0.0 50.0 100.0 250.0	0.76 ± 0.77 1.15 ± 1.44 0.56 ± 0.67 1.32 ± 1.64	0.0 50.0 100.0 200.0	0.43 ± 0.22 1.03 ± 0.74 0.93 ± 0.05 0.12 ± 0.01	0.0 50.0 100.0 300.0	2.89 ± 1.16 5.17 ± 3.21 3.77 ± 1.36 3.39 ± 3.35

Numbers indicate means ± SD of results obtained in a representative experiment with two cultures per experimental point (100 cells were evaluated per culture). 25 µM H_2_O_2_ was included as a positive control in each experimental series, for experiments with HepaRG 50 µM were used. Significant positive results were obtained with H_2_O_2_ in all experiments (data not shown). Acute toxicity was determined in each experiment with the CASY^®^ cell counter and analyzer system; the viability was in all experiments ≥ 70% (data not shown). Stars indicate statistical significance (Dunnett test; *P* value ≤ 0.05); n.d.: not determined

**Fig. 1:**
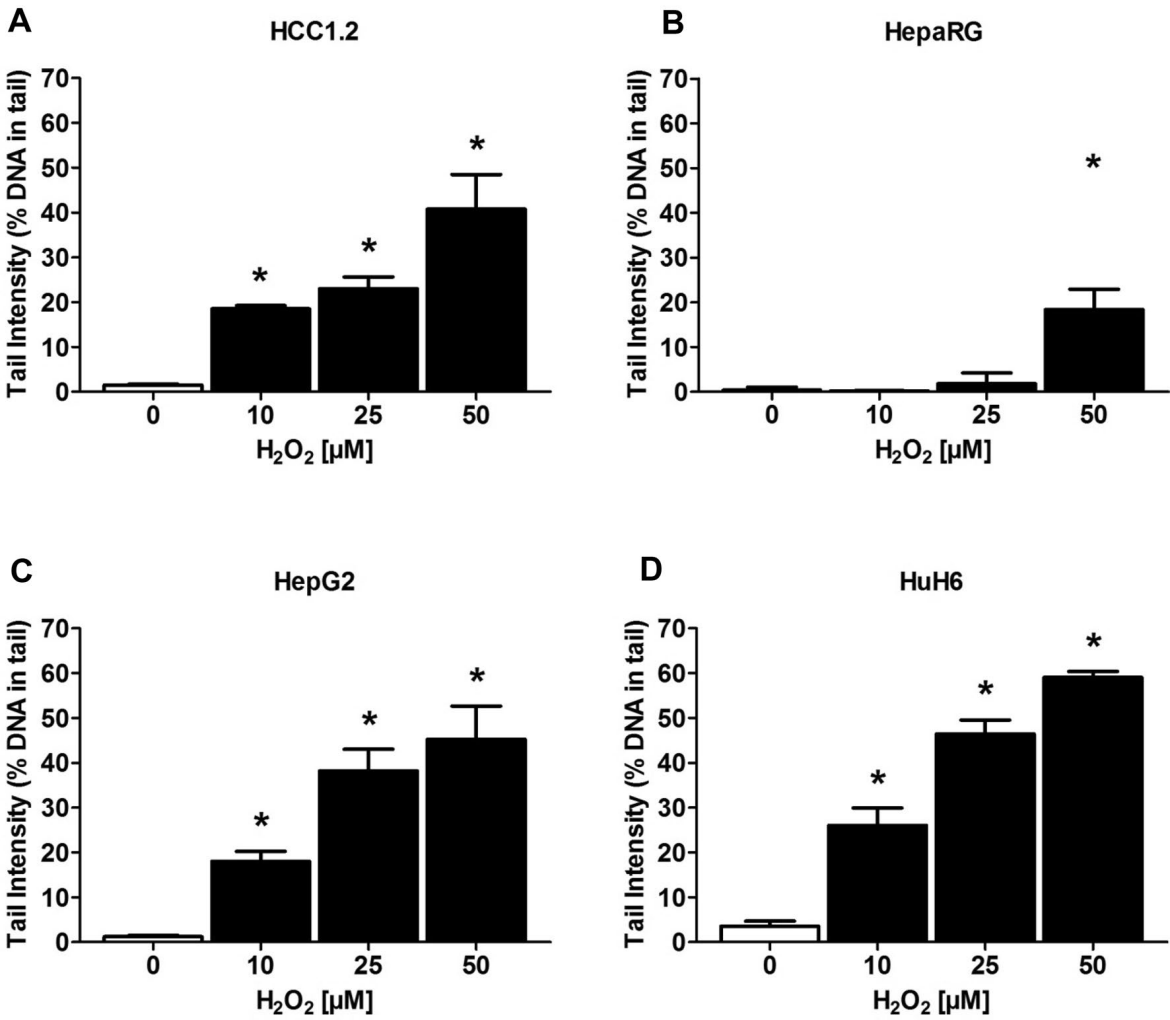
Induction of DNA damage in different human-derived liver cell lines by H_2_O_2_. Bars indicate mean ± SD of medians of four measurements (per experimental point in total 200 cells), asterisks indicate statistical significance (Dunnett’s Multiple Comparison Test, *P* ≤ 0.05)

H_2_O_2_ was used in all experiments as a positive control. We observed strong differences of the sensitivity of the different cell lines towards this peroxide. The results which were obtained in subsequent dose response experiments with selected cell lines are summarized in Fig. 1. Interestingly, HepaRG cells were by far less sensitive as HepG2, HuH6, and HCC1.2.

### Reproducibility of HuH6 experiments

**Fig. 2:**
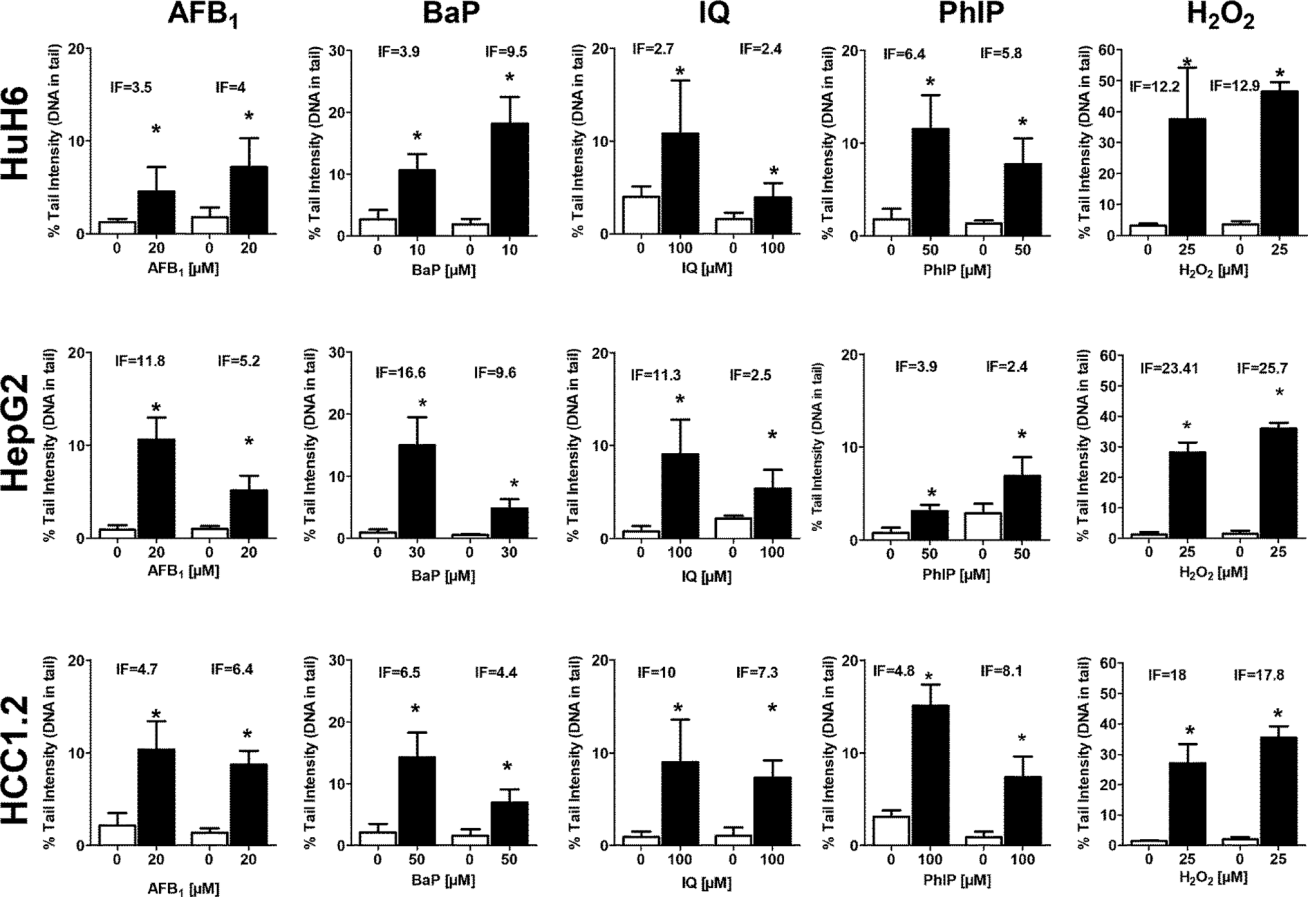
Reproducibility of SCGE experiments with different model mutagens in hepatoma cell lines. The cells were treated with different concentrations of the model compounds either for 24 h (AFB_1_, B(a)P) or for 48 h (IQ and PhIP), and H_2_O_2._ IF values represent the ratio of chemically induced comet formation (% DNA in tail) vs. DNA migration in corresponding controls. Bars show the results of two separate experiments; for each experimental point, at least two different cultures were set up in parallel, one slide with two gels was made per culture and 100 cells were counted per slide. Bars indicate mean ± SD of medians of four measurements; asterisks indicate statistical significance (Student *T* Test, *p* ≤ 0.05)

The reproducibility of results obtained with HuH6, HepG2, and HCC1.2 cells with different diagnostic mutagens in two independent experiments is depicted in Fig. 2. The reproducibility of results obtained with HuH6, HepG2, and HCC1.2 cells with the different model mutagens in two independent experiments is depicted in Fig. 2.

Induction factors (IF) represent the ratios of %DNA in tail induced in chemically treated cells and respective untreated controls. It can be seen that the values obtained in two independent experiments with different mutagens are in general similar in experiments with HuH6 and for certain model compounds (AFB1, H_2_O_2_ and IQ) also in assays with HCC1.2 while relatively strong differences of the responses were observed with HepG2. Experiments with HepaRG were not included as the reproducibility of comet experiments was addressed already in a study by Le Hegarat et al. (2014).

## Discussion

The results of the SCGE experiments confirm our assumption that several human-derived hepatoma cell lines detect the genotoxic properties of different groups of DNA reactive carcinogens without addition of exogenous enzyme activation mix. Seven out of twelve lines were sensitive towards AFB_1_, six detected B(a)P, five NDMA, four PhIP, and three IQ. Three hepatic lines (HepG2, HuH6, and HCC1.2) were sensitive towards representatives of all groups, HepRG to four compounds, HuH7 to two; only four lines were not responsive at all.

The sensitivity of the different hepatic lines was in general similar. The most pronounced effects were observed in general with AFB_1_ and B(a)P, which were positive at concentrations between 5 and 10 µM while higher doses of IQ and NDMA (in most cases ≥ 100 mM) were required to cause significant effects. It is notable that HCC1.2 was clearly more sensitive towards these latter compounds as the other lines.

The most important finding of the present study is the identification of a hepatic line (HuH6) which is apparently more suitable for the detection of genotoxins as the other liver-derived lines. This line was isolated more than 40 years ago from a hepatic tumor of a 1-year-old boy (Doi 1976). HuH6 cells have never been used in genotoxicity studies before according to our knowledge, however, some articles have been published concerning the impact of drugs on programmed cell death and cell signaling (Canal et al. 2015; Soini et al. 2017); furthermore, some data are available which concern the expression of drug metabolizing enzymes (i.e., CYP3A4, CYP3A7, CYP4F2, CYP4F3 and CYP2B6, and UGT1A1) in this line (Goldstein et al. 2013; Hosomi et al. 2011; Sugatani et al. 2010). In the present investigation, we obtained positive results with all test compounds; as shown in Fig. 2, these results were highly reproducible. The line has an intact and inducible *p53* and an epithelial morphology, its chromosome number is in a narrow range and twice as high as in primary hepatocytes; the doubling time is similar to that of most other lines.

Also HepG2 and HCC1.2 detected all five diagnostic genotoxins in the present experiments. The sensitivity of HepG2 can be explained by representation of a broad variety of drug metabolizing enzymes (Donato et al. 2013; Mersch-Sundermann et al. 2004); with HCC1.2, no results of enzyme measurements have been published, but gene expression analyses indicate that various phase I and II enzymes are present in this line (Winter et al. 2008). Our findings with HCC1.2 are in agreement with earlier findings (Winter et al. 2008). However, we found in the present study that these cells have an abnormal and unstable karyotype (i.e., 111–127 chromosomes) and a mutated *p53*. These findings indicate that they are not suitable for routine testing of chemicals; as mentioned above, it was stressed by Fowler et al. (2012) that *p53* competent cell lines are less prone to give false results as cells with mutated *p53*.

With HepG2 cells, clear positive results were obtained with all reference compounds in the present study. These results are in agreement with earlier reports (Knasmuller et al. 2004; Winter et al. 2008). However, the main problem of the use of this line is the poor reproducibility which is evident when results of experiments from different groups are compared in which similar or identical experimental concentrations were used (Knasmuller et al. 2004). Comparison of the LOEC values which were obtained in different laboratories show strong variations. For example, the concentration of AFB_1_ which was required to induce significant DNA migration was in previous experiments under identical conditions 20-fold lower as in the present study (Winter et al. 2008). Also the levels of B(a)P which caused positive results varied strongly in different labs (Uhl et al. 2000; Valentin-Severin et al. 2003). Another reason for this phenomenon may be strong fluctuations of the expression of genes encoding for drug metabolizing enzymes (Wilkening et al. 2003). Figure 2 concerns the reproducibility of comet experiments with different cell lines. It is evident that the results which were obtained with HepG2 vary over a relatively broad range, while results with HuH6 were in both experimental series similar. The reproducibility of experiments with HepaRG are described in a paper of Le Hegarat et al. (2014), the extent of comet formation which was observed in several independent experiments with cyclophosphamide was similar. Further information concerning the reproducibility of assays with HCC1.2 cells can be found in the publication of Winter et al. (2008). It is interesting that we found in the present study similar effects in independent experiments with hydrogen peroxide but fluctuations were observed with promutagens (which require metabolic activation). This indicates that the differences are probably due to instability of activities of drug metabolizing enzymes.

Recently, it was suggested that HepaRG cells, which are only commercially available, have a unique potential to detect genotoxins (Josse et al. 2012; Le Hegarat et al. 2014) and it was also stressed that these cells represent a reliable surrogate to human hepatocytes as they express high levels of phase I and II enzymes (Aninat et al. 2006; Antherieu et al. 2010). In the present study, HepaRG cells detected only four of five promutagens (AFB_1_, B(a)P, PhIP, and NDMA) and their sensitivity was in most cases identical or lower as that of the other cell types (HCC1.2, HepG2 or HuH6). With the heterocyclic aromatic amine IQ, negative results were obtained under all experimental conditions. These observations are in agreement with previous findings of Lehegerad et al. (2010) in SCGE and micronucleus experiments. They may be due to lack of metabolic activation of this heterocyclic aromatic amine which is catalyzed by CYP1A and *N*-acetyl transferase (NAT) (IARC 1993; Turesky et al. 1998). The latter enzyme is also essential for the activation of aromatic amines (Chevereau et al. 2017) which are an important group of genotoxic carcinogens (Vineis and Pirastu 1997). In this context, it is notable that negative results were obtained in SCGE experiments with HepaRG cells with two representatives of this group (2-acetylaminofluorene and 2,4-diaminotoluene) (Le Hegarat et al. 2014); data for di-nitro-PAHs (another relevant group of environmental mutagens) which require activation by NAT (Rothman et al. 1996; Talaska et al. 1996) are not available according to our knowledge. The cells have not been characterized in regard to their NAT activities, and results of gene expression analyses (RT-qPCR) showed that the expression of genes encoding for NAT1/NAT2 is not affected by HAAs (Dumont et al. 2010). Another disadvantage of HepaRG cells is their insensitivity towards induction of DNA damage by reactive oxygen species. As shown in Fig. 1, these cells were by far less responsive towards H_2_O_2_-induced DNA damage as the other lines (HepG2, HuH6, and HCC1.2). It is well documented that ROS play a crucial role in the induction of DNA damage by a large number of chemicals including peroxides and PAHs and also by radionuclides (Knasmuller et al. 2008; Ward et al. 1987).

An ideal cell type for the detection of genotoxic carcinogens would be primary human hepatocytes. However, it is difficult to obtain these cells (in particular from healthy individuals) and they are not available for routine testing. We found only two papers concerning comet assays with primary liver cells. The first by Monteith and Vanstone (1995) describes results which were obtained with B(a)P. An increase of DNA migration was found after treatment of the cells (for 3 h) with 50 µM. This concentration is similar to the dose which was required to induce significant DNA migration in the different hepatoma lines in the present study; however, the results were statistically not analyzed. Wilkening et al. (2003) compared the sensitivity of HepG2 and primary hepatocytes towards B(a)P, PhIP and DMN. They found no difference towards B(a)P, with the nitrosamine and the HAA substantially stronger effects were observed in the primary cells. The increased sensitivity of the hepatocytes is probably due to higher activities of drug metabolizing enzymes (in particular of cytochrome P450 isozymes which are involved in the activation of promutagens). Westerink and Schoonen (2007a, b) analyzed comparatively the levels of the phase I and phase II enzymes in human hepatocytes and HepG2, and found substantially higher activities in the primary cells.

Taken together, the results of the present study show that several human-derived liver cell lines detect promutagens without addition of rodent-derived liver homogenate. Furthermore, they indicate that HuH6 is the most promising line which may be useful for routine testing of chemicals. Results of experiments with HepaRG cells indicate that differentiated cells express higher levels of drug metabolizing enzymes (Aninat et al. 2006; Kanebratt and Andersson 2008); therefore protocols for genotoxicity assays with such cells were developed (Josse et al. 2012; Le Hegarat et al. 2010, for reasons of comparison, we also used differentiated HepaRG in the present experiments). Another possibility to improve the sensitivity and possibly also the reproducibility of experiments is the development of three dimensional models. It was recently suggested that 3D growth of HepG2 cells in spheroids is more suitable for cytotoxicity screening of chemicals (Ramaiahgari et al. 2014). Furthermore, preliminary findings of SCGE experiments with these cells indicate that growth in spheroids makes the cells more sensitive towards B(a)P as a consequence of increased levels of CYP1A1 (Shah et al. 2015). The possibility to increase the sensitivity and reproducibility of experiments with Huh6 by optimization of the cultivation conditions and of the exposure time is currently explored. Further experimental work with compounds that give false positive results in conventional in vitro tests as well as the characterization of drug metabolizing enzymes to confirm this assumption is also in progress.

## Electronic supplementary material

Below is the link to the electronic supplementary material.

Supplementary material 1 (DOCX 5223 kb)
