# Supplementary material for: Use of HuH6 and other human-derived hepatoma lines for the detection of genotoxins: a new hope for laboratory animals?
Source: Arch Toxicol. 2017 Dec 7;92(2):921–34. doi: 10.1007/s00204-017-2109-4 (PMC5818615; doi:10.1007/s00204-017-2109-4)

**Supplementary figures**

**Figure S1:** Morphological characteristics of the different cell lines. Photographs were made by use of an inverted research microscope (Eclipse Ti S, Nikon, Japan, magnifications 20 x).

**Figure S2A and 2B:** Growth characteristics of the different cell lines. 5.0x10^5^ cells were seeded into Petri dishes (Ø 6 cm) and cultivated over a period of 6 days. The cell numbers were determined by use of a CASY Cell Counter and Analyzer System in 24 h intervals. Each point represents the means ± SD of results obtained with three plates per experimental point. The doubling times were calculated using a non-linear fit with exponential growth equation (least squares fit).

**Figure S3:** Representative karyogram of HuH6. Per cell line 50 mitoses were analyzed as described in Materials and Methods.

**Figure S4:** Results of Western blot analyses of human derived liver cell lines. Untreated cells are indicated with C, B(a)P treated cells (30 µM for 24 h) are indicated with T. GAPDH was used as a reference protein.

**Figure S1**


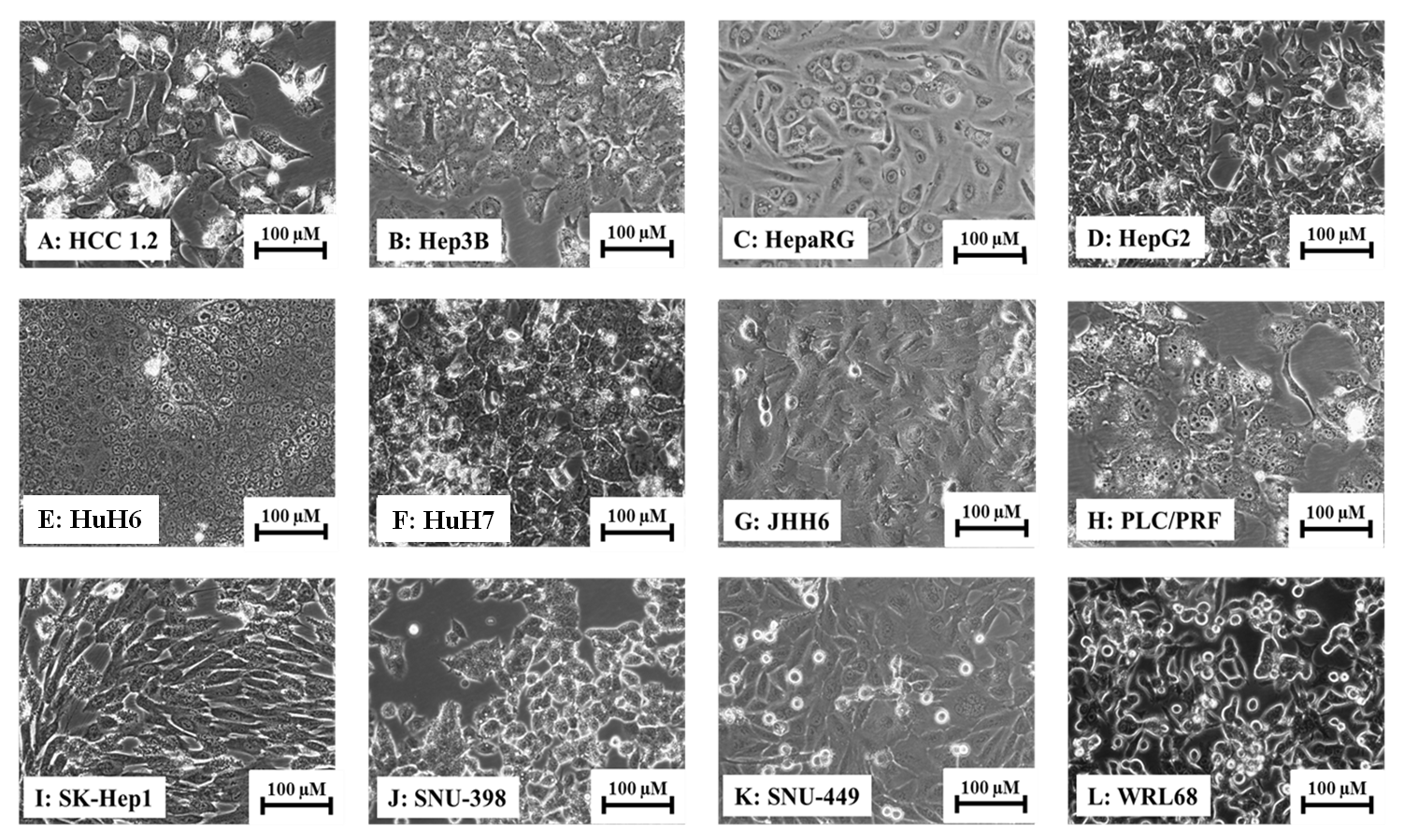


**Figure S2A**


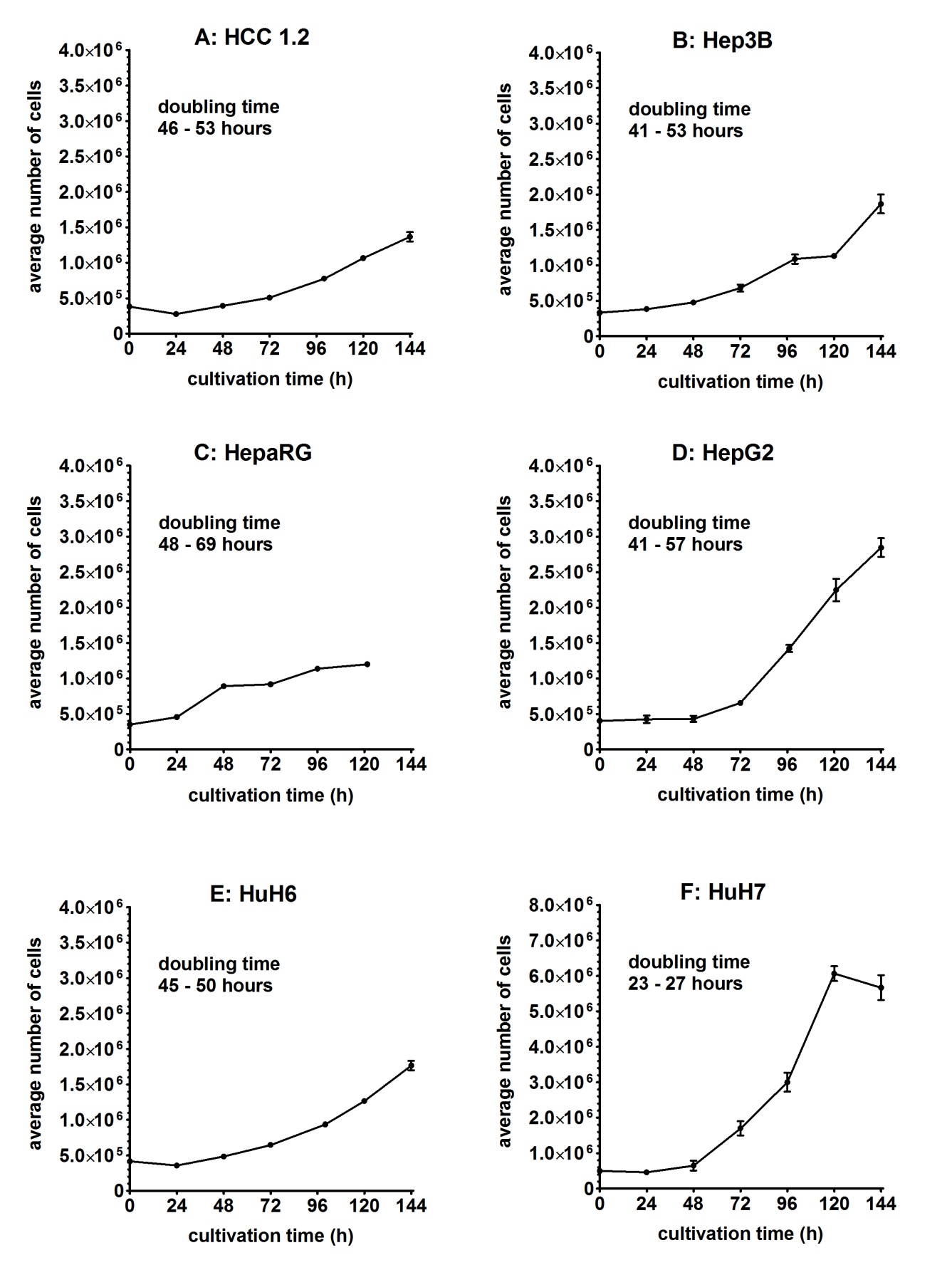


**Figure S2B**


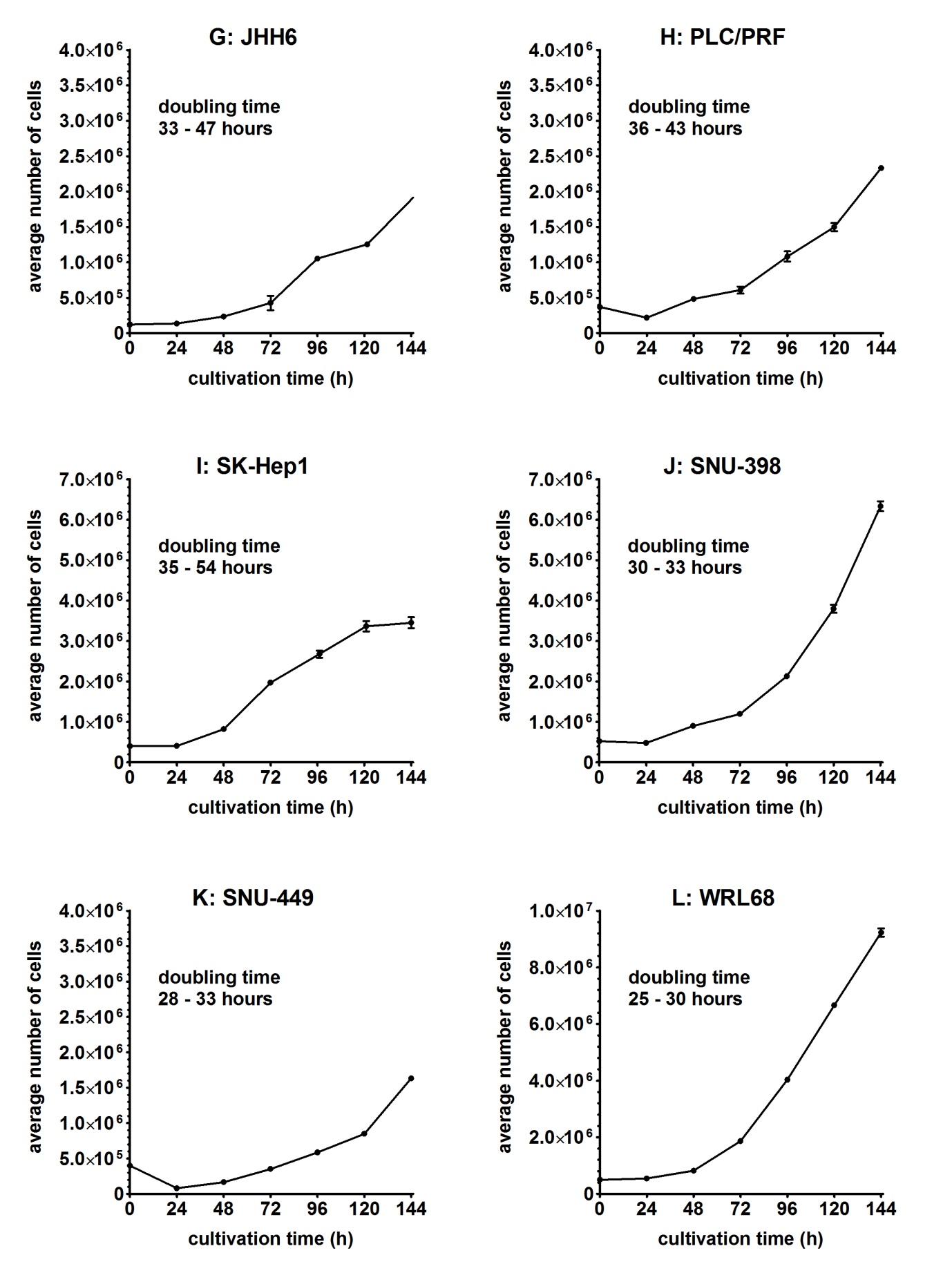


**Figure S3**


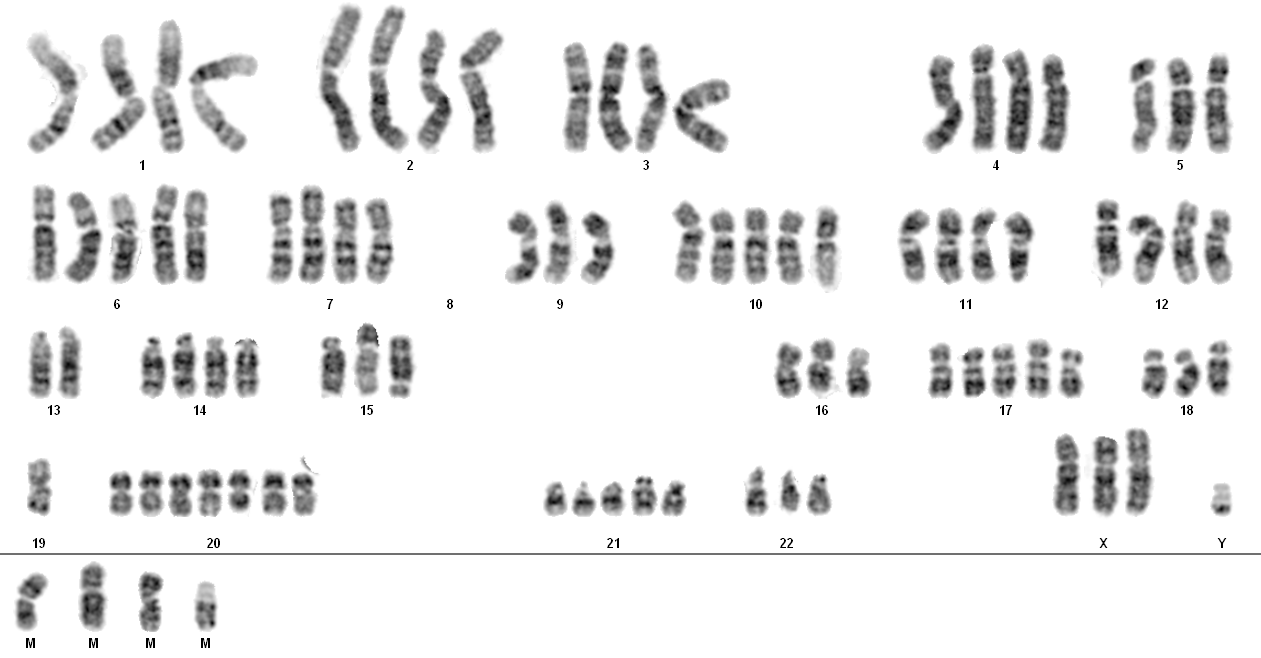


**Figure S4**


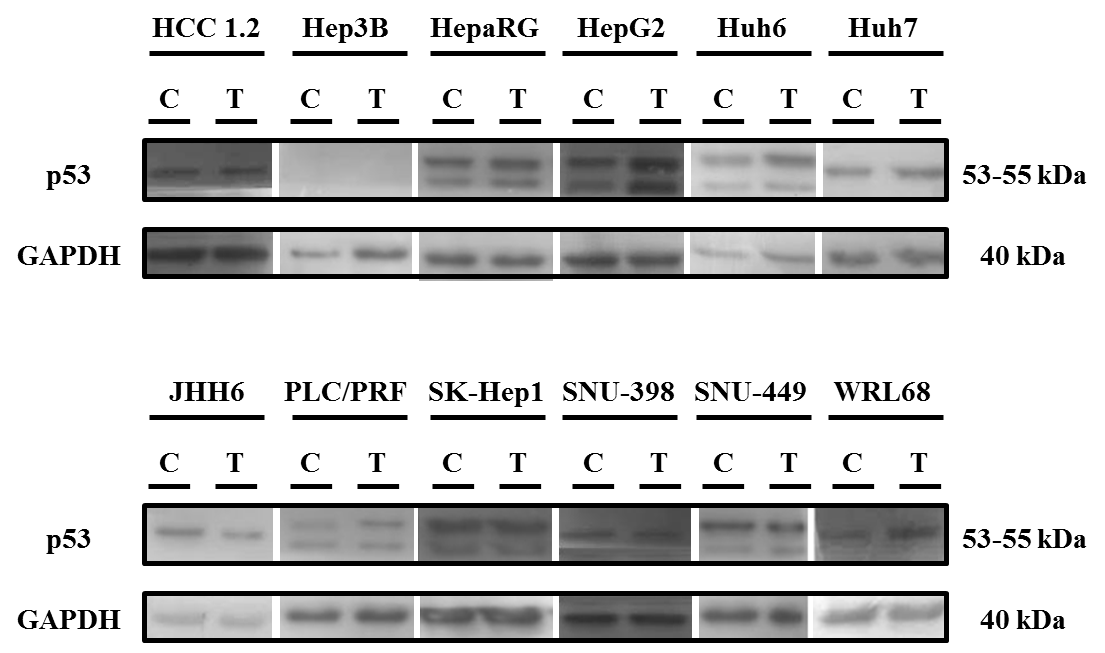

Supplement: Supplementary file 1 — Supplementary material 1 (DOCX 5223 kb) [file 204_2017_2109_MOESM1_ESM.docx]
